# The *ErChen* Decoction and Its Active Compounds Ameliorate Non-Alcoholic Fatty Liver Disease Through Activation of the AMPK Signaling Pathway

**DOI:** 10.3390/ph18111707

**Published:** 2025-11-11

**Authors:** Ye Wang, Yanting Liang, Man Hei Cheung, Xinran Wang, Huimei Mo, Jiehua Gan, Wei Yang, Jianmin Guo, Chun Liang

**Affiliations:** 1 Division of Life Science and State Key Lab for Molecular Neural Science, The Hong Kong University of Science and Technology, Hong Kong 999077, China; ywangij@connect.ust.hk (Y.W.); yliangas@connect.ust.hk (Y.L.); cheungmh@ust.hk (M.H.C.); xwanggg@connect.ust.hk (X.W.); ywdoccn@163.com (W.Y.); jmguo@ust.hk (J.G.); 2 EnKang Pharmaceuticals (Guangzhou), Ltd., Guangzhou 511455, China; mohuimei@intelgen.com.cn (H.M.); ganjiehua@intelgen.com.cn (J.G.); 3 Guangzhou Bay Area Institute of Biomedicine, Guangdong Lewwin Pharmaceutical Research Institute Co., Ltd., Guangdong Provincial Key Laboratory of Drug Non-Clinical Evaluation and Research, Guangzhou 510990, China

**Keywords:** *ErChen* decoction, NAFLD, insulin resistance, TCM, AMPK

## Abstract

**Backgrounds:** Non-alcoholic fatty liver disease (NAFLD) is a multifaceted metabolic disorder that has become a prominent public health problem worldwide. As a traditional Chinese medicine formula, the *ErChen* decoction (ECD) possesses significant effects on metabolic syndrome. **Methods:** To determine whether ECD can relieve lipid accumulation and insulin resistance (IR) in liver cells, NAFLD and IR cell models were established by treating HepG2 cells with free fatty acids and an overdose of insulin, respectively. Bioinformatics and experimental evidence demonstrated that ECD could ameliorate NAFLD by modulating multiple pathways. The optimal combination of the key compounds in ECD was identified by the orthogonal experiment. **Results:** For lipid homeostasis, ECD suppressed de novo lipogenesis and reduced the cholesterol level by activating the AMPK signaling pathway. Concurrently, ECD enhanced hepatic β-oxidation by inducing PPARα-mediated upregulation of ACOX-1 and CPT-1α. ECD also resolved hepatic insulin resistance by activating the IRS1-Akt-FoxO1 pathway. The combined treatment with 100 μM liquiritin (LQ), 200 μM glycyrrhizic acid (GA) and 200 μM hesperidin (HEN) exhibited the best effect in reducing TG content in NAFLD model cells. **Conclusions:** ECD exhibited superior activities in activating the AMPK signaling pathway compared to the optimal compound combination. The comparison between the ECD and its key compounds demonstrated the superior synergistic effects of the herbs in ECD.

## 1. Introduction

Non-alcoholic fatty liver disease (NAFLD) is defined as a metabolic imbalance condition due to excessive lipid accumulation in the liver, which occurs independently of heavy alcohol intake. The “multiple-hit hypothesis” of NAFLD states that lipid accumulation and insulin resistance (IR) in hepatocytes are considered the “first-hit” [1]. Consequently, the liver is exposed to many risk factors, such as oxidative stress and inflammation. In addition, adipose tissue dysfunction, nutritional factors, and gut microbiota all contribute to the aggravation of hepatic injury [1]. It has been shown that approximately 25% of the global population and 27% of China’s urban population are affected by NAFLD [2]. An analysis model based on published estimates and expert consensus projected that the total number of NAFLD patients will increase by 30% before 2030, especially in countries with rapid development [3]. Therefore, efforts to advocate a healthy diet, exercise and better treatment are needed.

At present, no specific drugs have received regulatory approval for the treatment of NAFLD. Optimal clinical management aims to ameliorate hepatic steatosis while preventing inflammatory progression and severe complications. Metformin is broadly prescribed for most type II diabetes (T2DM) patients for the improvement of hepatic glucose metabolism and increased peripheral glucose utilization [4]. Peroxisome proliferator-activated receptors (PPAR) γ agonists, like Pioglitazone, exhibit therapeutic potential by promoting fatty acid oxidation and improving insulin sensitivity in adipose and hepatic tissues [5]. In addition, evidence also reveals that statins and PPARα agonists (e.g., fibrates) may ameliorate concurrent hyperlipidemia and hepatic lipid accumulation through distinct mechanisms [6]. Since the frequent coexistence of NAFLD, IR, obesity, and T2DM, therapeutic strategies targeting single metabolic pathway demonstrate limited efficacy [7].

In traditional Chinese medicine (TCM) theory, it is believed that high-calorie diets, emotional disorders, and imbalances between work and relaxation disrupt the regulation of *Qi* by the liver in NAFLD patients. The weakness of the spleen and kidney systems further exacerbates dysregulation of the body functions and causes accumulation of “phlegm”, “dampness”, “turbidity” (in TCM theory, the approximate meanings of “phlegm” and “dampness” are unbalanced body liquid and lipid metabolism and the resulting complications), “blood stasis” (referring to high blood viscosity) and “heat” (referring to inflammation and the resulting complications) in NAFLD patients’ bodies. “Phlegm” and “dampness” will in turn impair the functions of the liver and establish a vicious circle [8]. According to *Tai Ping Hui Min He Ji Ju Fang* (Prescriptions of the Peaceful Benevolent Dispensary), the *Erchen* decoction (ECD) is a TCM formula consisting of Pinellia ternata (Thunb.) Breit. (PT), Citrus reticulata Blanco (CR), Poria cocos (Schwan.) Wolf. (PC), Glycyrrhiza uralensis Fisch. (GU), Zingiber officinale (Willd.) Rosc. (ZO) and Prunus mume (Sieh.) Sieb. et Zucc. (PM). Clinically, ECD can promote blood circulation, transform stasis and eliminate “phlegm”, thereby relieving NAFLD [8]. Previous phytochemical studies have identified key bioactive constituents in ECD, including flavonoids (e.g., liquiritin (LQ) and hesperidin (HEN), saponins and phenolics (e.g., 6-gingerol), which are considered the primary material basis for ECD’s therapeutic effects on metabolic disorders [9]. In addition, data from in vivo experiments indicated that ECD could ameliorate NAFLD by restoring intestinal barrier function and attenuating liver inflammation through the reduction in lipopolysaccharide translocation [8]. Nevertheless, limited research has been conducted on the molecular mechanisms of ECD in relieving NAFLD and IR.

To comprehensively and systematically identify potential targets associated with NAFLD and IR that can be affected by the ECD formula and its bioactive compounds, network pharmacology was employed to explore drug effects and interactions with multiple targets of NAFLD and IR. Validation experiments were conducted to corroborate the predicted molecular pathways derived from the network pharmacology analysis. Finally, orthogonal design analysis was employed to find the optimal combinations of the key compounds in ECD for the treatment of NAFLD. This research aimed to investigate the functions and molecular mechanisms of ECD and its key compounds in treating NAFLD, providing supporting evidence for its clinical application.

## 2. Results

### 2.1. Determination of the Chemical Profile of ECD Using HPLC

**Figure 1:**
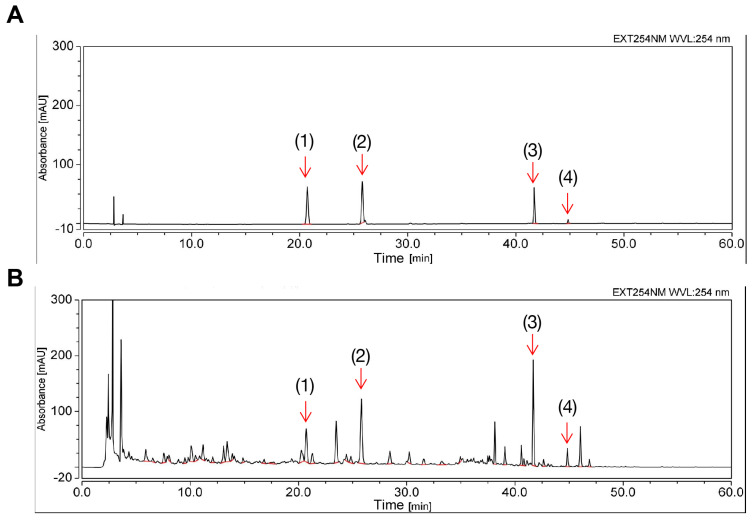
Chemical profile of ECD extract by HPLC. (**A**) Chromatogram results of mixed standard compounds. (**B**) Chromatogram results of the prepared ECD extract sample. The peaks of the following compounds are marked: (**1**) liquiritin (**2**) hesperidin (**3**) glycyrrhizic acid (**4**) 6-gingerol.

The fingerprint of ECD extract at an absorbance of 254 nm is shown in Figure 1. Several single compounds were identified, including HEN, LQ, glycyrrhizic acid (GA) and 6-gingerol. HEN is considered the standard substance of ECD, and its content was estimated to be 5.63 mg (HEN)/g (ECD extracts) by evaluating the peak area and the calibration curve (Supplementary Figure S1), in compliance with the requirements of the Pharmacopoeia of the People’s Republic of China.

### 2.2. ECD Reduced Lipid Accumulation in HepG2 NAFLD Cells

**Figure 2:**
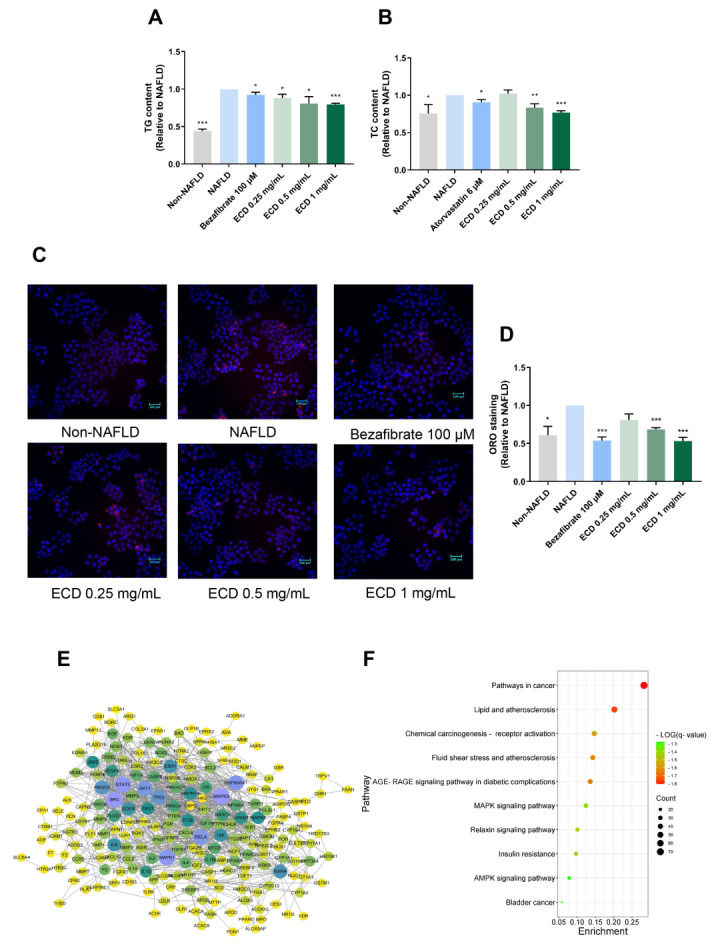
The effects of ECD in reducing lipid accumulation in HepG2 NAFLD model cells and network pharmacology results. (**A**,**B**) The TG and TC contents were reduced in HepG2 NAFLD model cells after treatment with ECD, represented as fold changes relative to the NAFLD model cells. (**C**,**D**) Representative images and quantification of the fluorescence signals of ORO staining in HepG2 NAFLD model cells with or without ECD treatment. Scale bar = 100 μm. (**E**) The PPI network consisted of 21 nodes and 100 strings, wherein the nodes with larger sizes and deeper colors denote the higher magnitudes of connectivity. (**F**) The top 10 relevant molecular pathways in the KEGG analysis. Data are presented as the mean ± S.E.M. (*n* = 3). *, *p* < 0.05; **, *p* < 0.01 and ***, *p* < 0.001 vs. the NAFLD model.

The excessive hepatic lipid accumulation is a distinct characteristic of NAFLD. The steatosis cell model was established by treating HepG2 cells with an OAPA mix. We measured the TG and TC levels in NAFLD model cells treated with ECD to determine its lipid-lowering effects. The TG and TC contents were increased by 55.8% and 24.3%, respectively, compared to the non-NAFLD control cells (Figure 2A,B). Significantly, compared to the NAFLD model cells, the TG and TC contents were reduced after treatment with ECD, e.g., by 20.4% and 23.1%, respectively, by 1 mg/mL ECD (Figure 2A,B). The ORO staining also indicated that ECD reduced the lipid droplets in the NAFLD model cells (Figure 2C,D). Treatment with ECD did not affect the cell viability (Supplementary Figure S2). These results revealed that the ECD treatment mitigated hepatic steatosis in the HepG2 NAFLD model cells.

### 2.3. Network Pharmacology Study of ECD

Network pharmacology was employed to explore the potential mechanisms of ECD in relieving NAFLD and IR. A total of 129 bioactive compounds in ECD were identified, and 577 targets of these compounds were predicted using the TCMSP and SwissTargetPrediction databases. Detailed information was provided in Supplementary Table S1. An herb-compound-target network was established to show the interaction network of the ECD’s compounds with the targets (Supplementary Figure S3A). A total of 447 targets related to NAFLD and 1845 targets related to IR were obtained from the databases. A Venn diagram (Supplementary Figure S3B) shows the 258 intersecting targets between the ECD targets and disease targets. Subsequently, a PPI network was constructed in light of the above targets to analyze their relationship (Figure 2E). The network contains 21 nodes and 100 strings, with each node’s size and color in the diagram representing the connections’ intensity. The top 20 targets with the highest degrees of connectivity are listed in Supplementary Table S2. Among the targets with high degrees of connectivity, RXRA, which can form a heterodimer with PPARα, and Akt are known to contribute to the metabolic balance of humans.

A bar chart and a bubble diagram were constructed to illustrate the functions of the hub targets, representing the results of GO and KEGG analyses, respectively. The top 10 results in three GO enrichment categories are shown in Supplementary Figure S3C. The results suggest that ECD may act on NAFLD and IR through several biological processes, including, e.g., cellular response to lipid, regulation of 5′ AMP-activated protein kinase (AMPK) cascade, and inflammation response. In the KEGG results (Figure 2F), several pathways were found to be potentially involved, such as lipid and atherosclerosis, AGE-RAGE signaling pathway in diabetic complications, insulin resistance, and AMPK signaling pathway, suggesting that these pathways are related to the underlying mechanisms of ECD in relieving NAFLD and IR. Since the PPARα and PPARγ have higher magnitudes of connectivity in the PPI network and herb-compound-target network, respectively, and Sterol Regulatory Element Binding Protein (SREBP) 1c is a transcriptional factor regulated by AMPK, molecular docking was performed to examine the potential interactions between the main compounds in ECD and the above targets. The results of binding energy (Supplementary Table S3) indicate robust binding affinities between the main compounds and the above hub targets. The binding sites and binding forces of HEN, LQ, GA, with hub targets, were further analyzed and visualized (Supplementary Figure S4), indicating the strong affinities between them.

### 2.4. ECD Activated AMPK and PPARα Signaling Pathways to Inhibit Lipid Synthesis and Promote β-Oxidation

**Figure 3:**
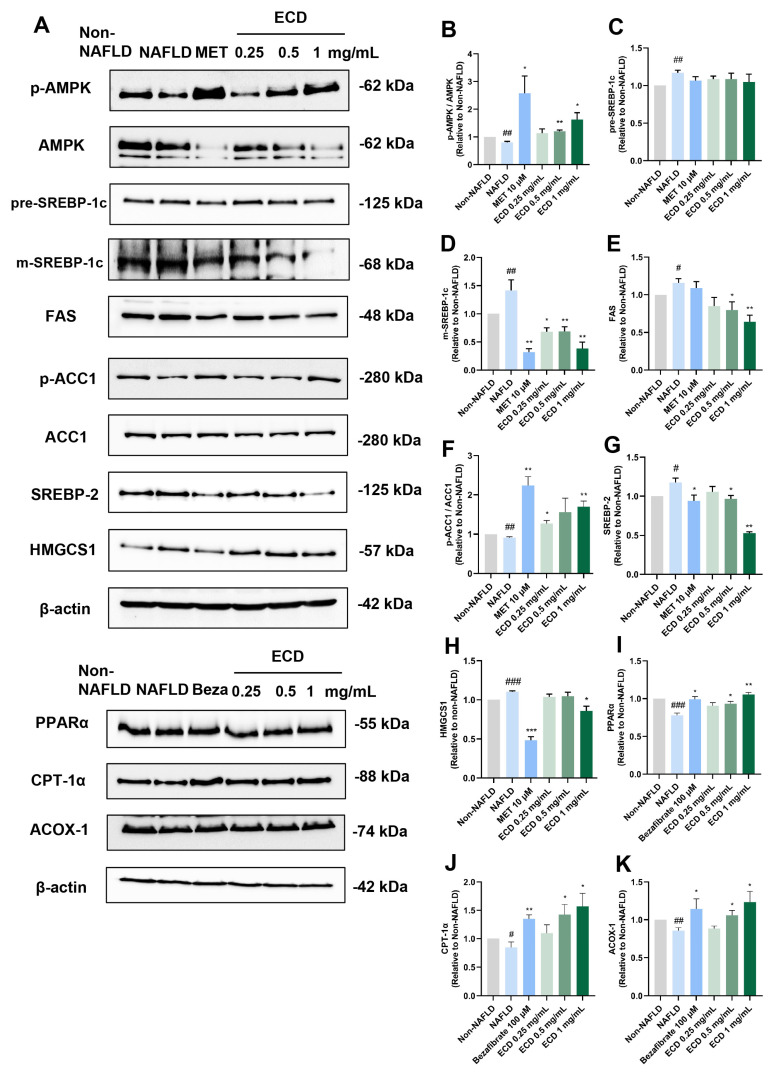
ECD activated AMPK and PPARα to inhibit lipid synthesis and promote β-oxidation of fatty acid in HepG2 NAFLD model cells. Target proteins in the AMPK and PPARα signaling pathways in HepG2 NAFLD model cells treated with ECD were analyzed by immunoblotting. (**A**) Representative immunoblots of the proteins in the AMPK and PPARα signaling pathways. (**B**–**K**) Quantification of the results of AMPK, SREBP-1c, FAS, ACC1, SREBP-2, HMGCS1, PPARα, CPT-1α and ACOX-1. Data are presented as the mean ± S.E.M. (*n* = 3) for each group. #, *p* < 0.05; ##, *p* < 0.01 and ### *p* < 0.001 vs. Non-NAFLD control. * *p* < 0.05 and ** *p* < 0.01 and vs. ***, *p* < 0.001 the NAFLD model.

To further confirm whether ECD can modulate the AMPK and PPARα signaling pathways as predicted from network pharmacology, we utilized immunoblotting to examine the protein level changes after the ECD treatment (Figure 3A). The results indicated that ECD treatment significantly activated AMPK by phosphorylation (Figure 3B). Activated AMPK can phosphorylate SREBP-1c at Ser372, thus the cleavage and nuclear translocation of mature type of SREBP-1c (m-SREBP-1c) and its target gene expression involved in de novo lipogenesis will be suppressed [10]. The immunoblotting results revealed that ECD treatment dramatically reduced the levels of m-SREBP-1c and its downstream protein, fatty acid synthase (FAS), but not the precursor type of SREBP-1c (p-SREBP-1c) (Figure 3C–E). ECD treatment also elevated the phosphorylation level of Acetyl-CoA carboxylase 1 (ACC1), making it inactive (Figure 3F). In addition, ECD treatment significantly reduced the levels of SREBP-2 and HMG-CoA synthase 1 (HMGCS1) involved in the cholesterol synthesis process (Figure 3G,H). To conclude, ECD treatment can activate AMPK, thereby inactivating ACC1 and reducing the protein levels of m-SREBP-1c, SREBP-2, FAS and HMGCS1, suggesting that ECD can inhibit endogenous lipid synthesis through the AMPK pathway in HepG2 NAFLD model cells.

PPARα can promote its downstream protein expression through recognizing and binding to PPREs to form a ligand-activated transcriptional complex [11]. This elevates the levels of the rate-limiting enzymes regulating peroxisomal β-oxidation, such as that of peroxisomal acyl-coenzyme A oxidase 1 (ACOX-1), and increases the expression of carnitine palmitoyltransferase 1α (CPT-1α), which triggers fatty acid transport across the mitochondrial membrane [12]. Thus, we determined the PPARα and its downstream target levels in HepG2 NAFLD model cells treated with ECD. The immunoblotting results revealed that the OAPA-induced reduction in PPARα, CPT-1α and ACOX-1 levels was increased by 35%, 78% and 44%, respectively, after ECD (1 mg/mL) treatment (Figure 3I–K). In conclusion, ECD activated the PPARα pathway to improve β-oxidation of fatty acids, thus protecting hepatocytes from lipid accumulation.

### 2.5. ECD Increased Insulin Sensitivity and Promoted Glucose Uptake in HepG2 IR Cells

NAFLD exhibits a strong correlation with IR in both hepatocytes and adipocytes, representing the initial trigger in NAFLD pathogenesis [1]. IR leads to inhibition of protein kinase B (Akt) activation, causing reduced phosphorylation of forkhead box protein O1 (FoxO1), making it active. FoxO1 is a transcription factor that promotes gluconeogenesis and de novo lipogenesis [13]. Hence, IR will deteriorate the state of NAFLD.

**Figure 4:**
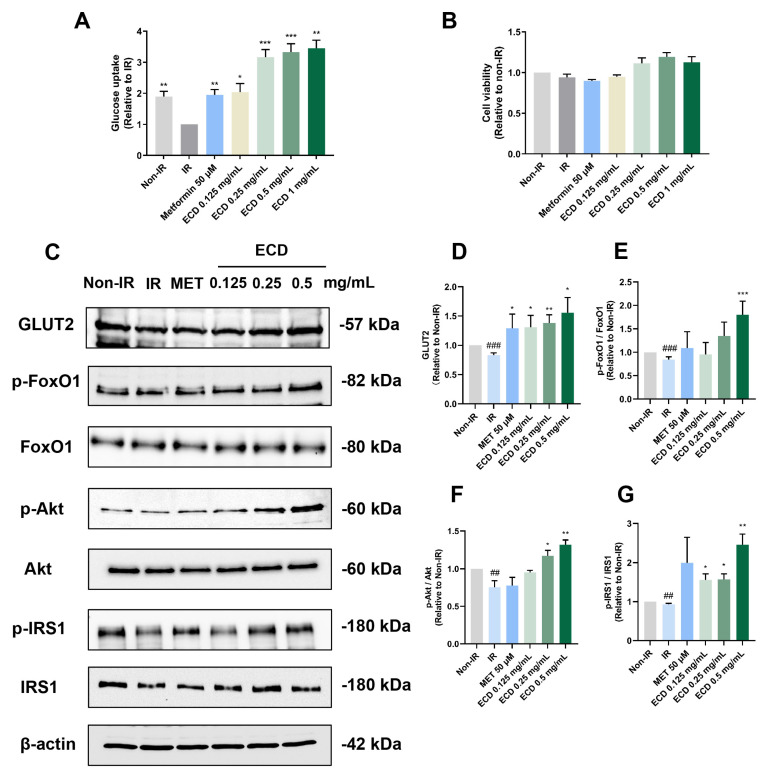
ECD relieved IR in HepG2 cells. HepG2 IR model cells were treated with ECD for 24 h. (**A**) The glucose uptake change was represented as a fold change relative to the IR group. (**B**) Cell viability was measured after being treated with insulin and Metformin or 0.125–0.5 mg/mL ECD. (**C**) Representative immunoblots of proteins related to insulin sensitivity and glucose transportation. (**D**–**G**) The level of GLUT2 (**D**) and the phosphorylation levels of FoxO1 (**E**), Akt (**F**) and IRS1 (**G**) were quantified. Data are presented as the mean ± S.E.M. (*n* = 3). ## *p* < 0.01 and ### *p* < 0.001 vs. the Non-IR control. * *p* < 0.05; ** *p* < 0.01 and *** *p* < 0.001 vs. the IR model.

To determine if ECD can alleviate IR and promote glucose uptake, the HepG2 IR cell model was established using an overdose of insulin [14], followed by measurement of glucose uptake in the HepG2 IR cells with and without ECD treatment. Compared to non-IR cells, glucose uptake was markedly decreased in HepG2 IR cells without ECD treatment, while ECD treatment could improve the ability of HepG2 IR cells to uptake glucose (Figure 4A) without causing obvious cytotoxicity (Figure 4B). Immunoblotting results showed that while the glucose transporter 2 (GLUT2) level decreased in IR cells compared to non-IR cells, the level of GLUT2 increased in IR cells treated with the positive control drug Metformin or ECD, to levels that were even higher than non-IR cells (Figure 4C,D). To further evaluate whether ECD could relieve gluconeogenesis, the most important characteristic of IR, the phosphorylation status of the key factors was determined. The FoxO1 activity was elevated by dephosphorylation in IR cells, whereas ECD could inactivate it by phosphorylation in a dose-dependent manner (Figure 4C,E). In IR cells, the activities of Akt and IRS-1 were inhibited by dephosphorylation, whereas the phosphorylation levels of Akt and IRS-1 increased by ECD treatment in a dose-dependent manner (Figure 4C,F,G), activating the insulin signaling pathway. These data suggest that insulin sensitivity is enhanced and gluconeogenesis is attenuated by ECD in the IR cells.

### 2.6. Inhibition of De Novo Lipogenesis by ECD Depends on AMPK Activation

**Figure 5:**
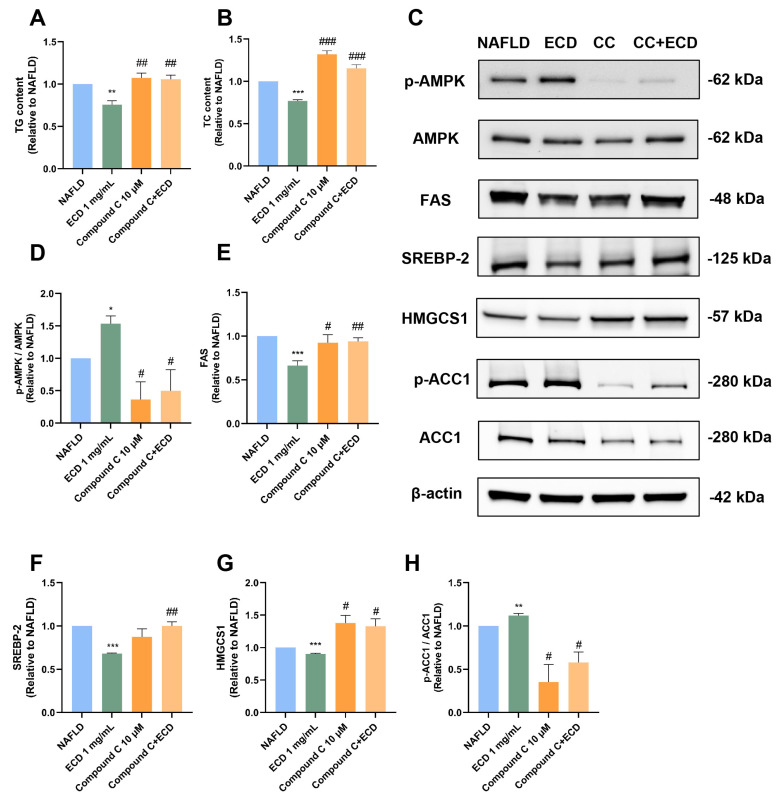
ECD inhibited de novo lipogenesis through the AMPK signaling pathway. HepG2 NAFLD model cells were treated with the solvent, ECD, CC or CC + ECD for 48 h. The TG (**A**) and TC (**B**) contents were measured and represented as fold change relative to the NAFLD model cells. (**C**–**H**) Immunoblots (**C**) and quantification of the levels and/or phosphorylation levels of AMPK (**D**), FAS (**E**), SREBP-2 (**F**), HMGCS1(**G**) and ACC1 (**H**). Data are presented as the mean ± S.E.M. (*n* = 3) for each group. * *p* < 0.05; ** *p* < 0.01 and *** *p* < 0.001 vs. the NAFLD model. # *p* < 0.05; ## *p* < 0.01 and ### *p* < 0.001 vs. ECD (1 mg/mL) group.

To confirm whether ECD treatment inhibits de novo lipogenesis by activating the AMPK signaling pathway, we examined the influence of an AMPK inhibitor, Compound C (CC), on HepG2 NAFLD model cells treated with ECD. As illustrated in Figure 5A,B, CC treatment counteracted the effects of ECD in reducing TG and TC accumulation in HepG2 NAFLD model cells. Immunoblotting results indicated that the phosphorylation levels of AMPK in the cells treated with CC or CC + ECD were notably lower than in the NAFLD and ECD groups (Figure 5C,D). Additionally, the inhibitory effects of ECD on FAS, SREBP-2, and HMGCS1 were attenuated upon inhibition of AMPK by CC in HepG2 NAFLD cells (Figure 5C,E–G). The inhibitory phosphorylation of ACC1, which was increased by ECD, was also reduced (Figure 5H). These data support that ECD inhibits the de novo lipogenesis through the AMPK signaling pathway.

### 2.7. Combined Treatment of HepG2 NAFLD Model Cells with LQ, GA and HEN Significantly Reduced De Novo Lipogenesis

**Figure 6:**
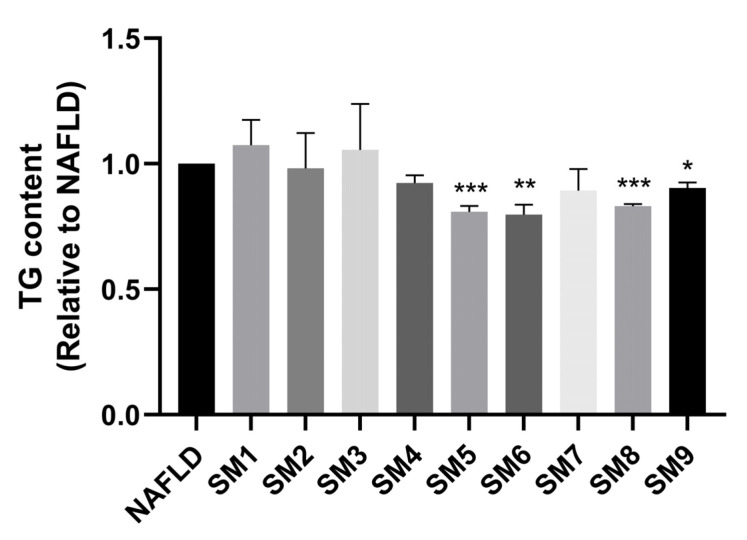
SM6 significantly reduced the TG accumulation in HepG2 NAFLD cells. HepG2 NAFLD model cells were treated with different combinations of compounds for 48 h. The TG content was measured and represented as fold change relative to the NAFLD group. Data are presented as the mean ± S.E.M. (*n* = 3). * *p* < 0.05; ** *p* < 0.01 and *** *p* < 0.001 vs. the NAFLD model.

According to the HPLC analysis, cell viability assay and TG and TC contents determination (Supplementary Figure S5), LQ, GA and HEN are considered to be the key active compounds in ECD. To determine the optimal combination of these compounds for the reduction in lipid accumulation, an orthogonal experiment was employed, in which the HepG2 NAFLD model cells were treated with the compound combinations as shown in methods 4.9. The TG content was chosen as an evaluation parameter for variance analysis. As shown in Figure 6, the TG accumulation in cells treated with different compound combinations was reduced to varying degrees, with the SM6 treatment group being the lowest. After calculating the K and R values as shown in Table 1, the optimal combination of A2B3C3, which corresponds to SM6 (100 μM LQ, 200 μM GA and 200 μM HEN), had the best effects in reducing TG accumulation in HepG2 NAFLD model cells. The orthogonal design variance analysis is illustrated in Supplementary Table S4. SM6 also demonstrated a synergistic effect in reducing TG accumulation. Detailed computational procedures are provided in Supplementary Table S5.

**Figure 7:**
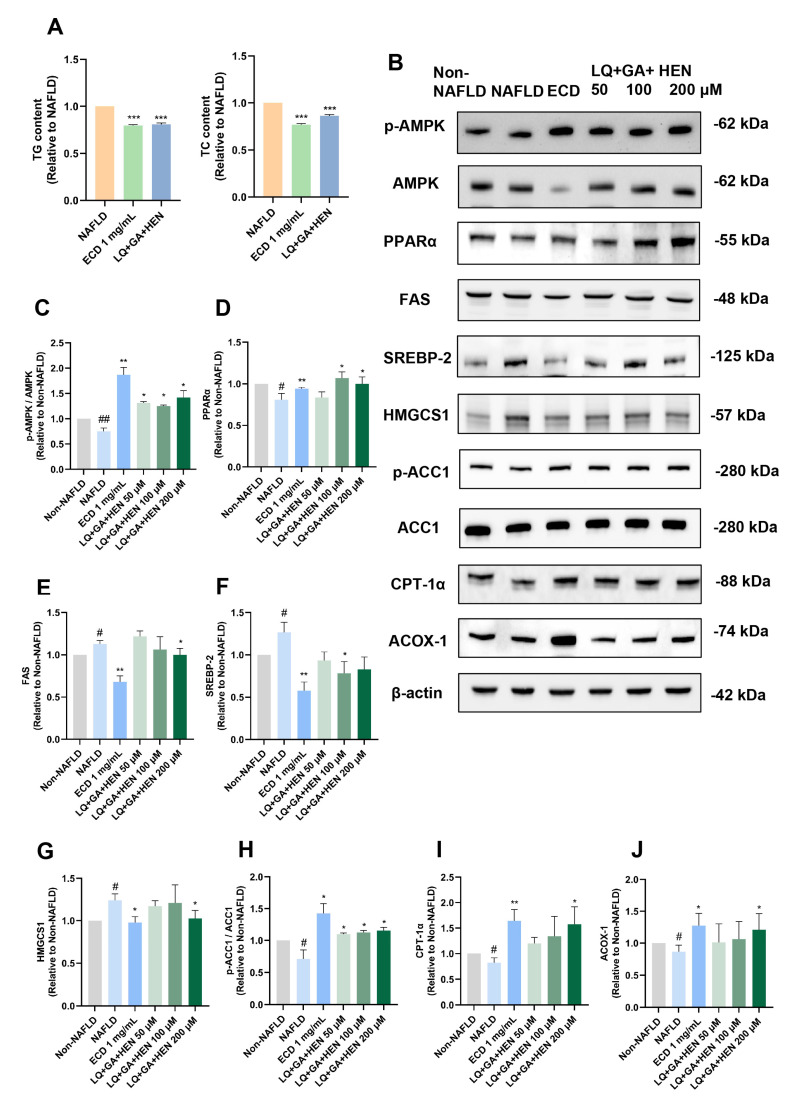
The comparison of the effects of SM6 and ECD in reducing lipid accumulation and activating AMPK and PPARα signaling pathways. (**A**) HepG2 NAFLD model cells were treated with the SM6 combination of the compounds or ECD for 48 h. The TG and TC contents were measured and represented as fold change relative to the NAFLD cells. (**B**) HepG2 NAFLD model cells were treated with different combinations of compounds or ECD for 48 h, followed by immunoblotting analysis of the key proteins in the AMPK and PPARα signaling pathway. (**C**–**J**) Quantification of the results of AMPK, PPARα, FAS, SREBP-2, HMGCS1, ACC1, CPT-1α and ACOX-1. Data are presented as the mean ± S.E.M. (*n* = 3) for each group. # *p* < 0.05; and ## *p* < 0.01 vs. Non-NAFLD control. * *p* < 0.05; ** *p* < 0.01 and *** *p* < 0.001 vs. the NAFLD model.

Our data also indicated that the SM6 had similar effects in reducing TG as 1 mg/mL ECD, but its impact in lowering TC (by 13%) was slightly inferior than 1 mg/mL ECD (by 23%) (Figure 7A). Immunoblotting results (Figure 7B) and the quantification revealed that the AMPK signaling pathway and PPARα signaling pathway were both significantly activated by the SM6 treatment (Figure 7C,D). SM6 reduced the protein levels of FAS, SREBP-2 and HMGCS1 (Figure 7E–G), and inactivated ACC1 (Figure 7H); however, its effects were not as good as 1 mg/mL ECD. In addition, the effects of SM6 in increasing the protein levels of PPARα, CPT-1α and ACOX-1 were similar to 1 mg/mL ECD (Figure 7D,I,J).

## 3. Discussion

Influenced by multiple factors, including hepatic lipid accumulation, IR, oxidative stress, inflammatory response, and so on, NAFLD is a metabolic disorder, often occurring in overweight and obese people without consuming excessive alcohol [1]. Apart from lifestyle improvements such as nutritional intervention and weight loss, there are limited strategies to alleviate NAFLD. As TCM is effective in the management of metabolic syndrome as an alternative and complementary treatment, it arouses scientists’ interest in researching the underlying mechanisms. Previous studies have demonstrated that ECD could ameliorate NAFLD in high-fat-diet induced mice by reducing inflammation [8] and affecting the gut microbiota [9]. Nevertheless, the research on the underlying molecular mechanisms is still incomprehensive. In the current research, network pharmacology analysis was performed, followed by cell-based experiments to verify the predictive results. We demonstrated that ECD and its compounds displayed high activities against lipid accumulation and IR in HepG2 cells.

Since the therapeutic principle of TCM drugs is complicated, network pharmacology is recognized as an effective strategy for investigating the multi-component and multi-target characteristics of TCM. KEGG analysis demonstrated that the potential targets of ECD are highly related to IR pathways and the AMPK pathway. AMPK serves a crucial function in balancing nutrient metabolism. In addition, from the PPI results, RXRA and PPARα both have high degree values, indicating that the activation of the RXRA/PPARα heterodimer is an essential mechanism for ECD. Therefore, we decided to conduct validation experiments focusing on these pathways to reveal the mechanisms of ECD in relieving NAFLD.

The complex and bidirectional relationship between NAFLD and IR requires a comprehensive investigation. Elevated free fatty acid (FFA) can reduce insulin’s ability to restrain hepatic glucose production because of the breakdown of autoregulation [15]. Meanwhile, hyperinsulinemia can also stimulate de novo lipogenesis via SREBP-1c. Here, we demonstrated that FFA-induced TG and TC accumulation in the HepG2 NAFLD model cells could be relieved by ECD treatment. Suppression of de novo lipogenesis could be achieved by ECD treatment through the activation of AMPK and ACC1, and the downregulation of m-SREBP-1c and FAS. In addition, the transfer of activated fatty acids on the mitochondrial membrane for oxidative degradation could be promoted by ECD, likely through the upregulation of CPT-1α. Moreover, the imbalance of cholesterol metabolism could be improved by reducing the SREBP-2 and HMGCS1 levels, which are involved in the vital steps of cholesterol synthesis.

Our current findings also demonstrate that ECD treatment can activate hepatic insulin signaling in HepG2 IR cells. When IR is alleviated, the insulin receptor activity will be increased, which can be measured by IRS1 phosphorylation [16]. Notably, ECD treatment activated IRS1 by phosphorylation at the Tyr895 site. Our results suggested that in ECD-treated HepG2 IR cells, the IRS1-Akt signaling pathway was activated, thus promoting the inhibition of FoxO1. This contributes to inhibiting gluconeogenesis and promoting glucose transport in HepG2 IR cells.

The accumulation of TG in liver cells is a vital parameter for evaluating the occurrence of NAFLD. Therefore, we used the TG content as an evaluation parameter to determine the optimal combination of the active compounds in ECD by orthogonal analysis. The results suggested that the combined treatment with LQ, GA and HEN exhibited different effects in lowering the TG content, with the SM6 combination (100 μM LQ, 200 μM GA and 200 μM HEN) showing the best effect. Immunoblotting results also revealed that SM6 significantly activated the AMPK and PPARα signaling pathways, and the effects in promoting the protein expression of PPARα, CPT-1α and ACOX-1 are similar to ECD. However, the concentrations of the three compounds in SM6 are much higher than their actual concentrations in the ECD extract, while the ECD formula demonstrated superior activities in reducing TC accumulation and activating the AMPK signaling pathway. These results demonstrate that the ECD formula has superior synergistic effects than the combination of main compounds in relieving NAFLD.

Our study contributes to the modernization of TCM by employing a systematic strategy including network pharmacology prediction, in vitro validation, and optimization of combined active compounds, to decipher the complex mechanism of a classic TCM formula. In the context of the current lack of approved pharmacotherapies for NAFLD, our work provides a scientific rationale for ECD as a promising multi-targeted therapeutic candidate for NAFLD. On the other hand, while this study provides mechanistic insights into the anti-NAFLD effects of ECD and its compounds using the HepG2 cell model, it is important to consider the inherent limitations that HepG2 cells exhibit metabolic profiles that differ significantly from primary hepatocytes. Notably, a comprehensive study by Nagarajan et al. demonstrated that HepG2 cells have an abnormally high rate of glucose incorporation and a reduced capacity for fatty acid β-oxidation [17]. Consequently, the potent effects on glucose and fatty acid metabolism induced by ECD observed in our HepG2 model warrant further investigation in primary cells and animals. In this regard, previous in vivo studies have demonstrated that ECD can effectively ameliorate NAFLD in animal models [8,9].

While this study demonstrates the efficacy and reveals some aspects of the molecular mechanisms of ECD, its translational potential requires further investigation into safety and pharmacokinetics. Future work will include in vivo safety assessments and pharmacokinetic profiling to address these crucial issues. Furthermore, supporting evidence from in vivo study is also needed in the future to demonstrate the significant amelioration of lipid accumulation and IR by ECD.

## 4. Materials and Methods

### 4.1. Chemicals and Reagents

The reference chemical standards used for HPLC identification and following experiments were Hesperidin (Purity ≥ 98%, NIFDC-110721, Beijing, China), Liquiritin (Purity ≥ 98%, Macklin Biochemical Technology Co., Shanghai, China, L886004), Glycyrrhizic acid (Purity ≥ 98%, Macklin, G810519, Shanghai, China), and 6-Gingerol (Purity ≥ 98%, Macklin, G810517). Acetonitrile (Chromatographic Pure) was purchased from SINENCE (Fuzhou, China. Methanol (AR) and Phosphoric acid (AR) were purchased from Tianjin Zhiyuan Chemical Reagent Co., Ltd. (Tianjin, China).

### 4.2. ECD Extract Preparation

The raw materials of the six single herbs in ECD are shown in Table 2. All herbs have been authenticated by Prof. Wei-min Li, a retired professor from the Guangzhou University of Chinese Medicine. The herbs were boiled and extracted in ten volumes/weight (10:1, *v*/*w*) of distilled water for 1 h first, and then in eight volumes/weight (8:1, *v*/*w*) for another 1 h. The extracts were combined, concentrated, and subjected to low-pressure rotary evaporation to prepare the ECD extract powder. The final product was dissolved in water to prepare a stock solution with a 10 mg/mL concentration. The ECD sample was sterilized by filtration through a 0.22-μm filter and stored at a temperature of 4 °C.

### 4.3. HPLC Identification of the Chemical Composition of ECD Extract

The chemical profile of ECD was analyzed using HPLC (UltiMate™ 3000 Standard HPLC Systems, Thermo Fisher Scientific, Waltham, MA, USA). Chromatographic separations were conducted on a Phenomenex PS C18 column (5 μm). The flow rate was 1 mL/min, and the column temperature was 30 °C. A mobile phase system consisting of acetonitrile (A) −0.1% phosphoric acid in H_2_O (B) was used with the following gradient process: 0–30 min, 10–27% A, 90–73% B; 30–60 min, 27–95% A, 73–5% B. The sample injection volume was 10 μL.

### 4.4. Network Pharmacology Analysis

The active compounds of the six herbs in ECD were selected by the following criteria: oral bioavailability (OB) ≥ 30%, drug-likeness (DL) ≥ 0.15 and Caco-2 cell permeability ≥ −0.4 in the TCMSP Platform (https://tcmsp-e.com/tcmsp.php, accessed on 3 August 2023 [18]. Compounds not documented in TCMSP were supplemented with references to relevant literature. The targets of ECD were gathered by using the TCMSP and SwissTargetPrediction (http://www.swisstargetprediction.ch/, accessed on 3 August 2023) [19] databases. “Non-alcoholic fatty liver disease” and “insulin resistance” were used as keywords to search for disease targets in the OMIM database [20] and the DisGeNet database (relevance score ≥ 0.01) [21]. The overlapped targets between ECD-related targets and disease-related targets were obtained and loaded into the STRING 11.5 database [22] for protein–protein interaction (PPI) analysis. In addition, the overlapped targets were imported to the Metascape platform [23] for gene ontology (GO) enrichment [24] and Kyoto Encyclopedia of Genes and Genomes (KEGG) pathway analysis [25].

### 4.5. NAFLD Model Cells and Cell Viability of ECD-Treated Hepatocytes

HepG2 was obtained from the American Type Culture Collection (ATCC) and cultured in Dulbecco’s modified Eagle’s medium (DMEM, Invitrogen, Carlsbad, CA, USA) containing 10% fetal bovine serum (FBS, Invitrogen, Carlsbad, CA, USA) at 37 °C with 5% CO_2_ [26]. HepG2 cells were treated with a mixture of oleic acid (OA, Sigma-Aldrich, St. Louis, MO, USA) and palmitate (PA, Sigma-Aldrich, St. Louis, MO, USA) (OAPA mix; 0.05 mM and 0.2 mM of OA for triglyceride measurement and cholesterol measurement, respectively; OA:PA = 2:1) for 24 h to establish the NAFLD model cells. The HepG2 cells were treated with ECD extract (0.25, 0.5, 1 mg/mL) for 48 h and the cell viability was determined by the WST-1 assay (Dojindo Molecular Technologies, Inc., Kumamoto, Japan).

### 4.6. Oil Red O Staining

HepG2 cells were seeded in 12-well plates at 5 × 10^5^ cells/well and cultured for 24 h. The cells were treated with the OAPA mix for 24 h with or without ECD (0.25, 0.5 and 1 mg/mL) or bezafibrate (100 μM; Sigma-Aldrich, USA) for 48 h. Subsequently, the cells were fixed with 4% paraformaldehyde for 30 min and then stained with a 0.3% oil red-O working solution for 15 min at room temperature. Afterwards, the stained cells were washed with PBS and 60% isopropanol. DAPI staining was performed for 30 min in the dark. The cells were observed by fluorescence microscopy (Nikon, Tokyo, Japan).

### 4.7. Measurement of Intracellular Triglyceride (TG) and Total Cholesterol (TC) Levels

The HepG2 cells were treated with OAPA mix and drugs as planned. The cells were harvested, and the TG and TC levels were determined using the corresponding assay kits (Applygen, Beijing, China). The protein contents were measured using a BCA Protein Assay kit (Applygen, China).

### 4.8. Quantification of Glucose Uptake

In the IR model, HepG2 cells were stimulated with 0.5 μM insulin (Thermo Fisher Scientific, USA) [14] for 24 h, then starved in DMEM without serum for 2 h, and further treated with ECD (0.125, 0.25, 0.5 mg/mL), or metformin (MET, 50 μM; Jingfeng Pharmaceutical Co. Ltd., Beijing, China), as a positive drug, for another 24 h. The culture medium was collected for the determination of the glucose content using a commercial kit (Applygen). Subsequently, WST-1 assay was conducted to measure cell viability.

### 4.9. Orthogonal Experimental Design

The orthogonal experimental design was employed to identify the optimal combination of LQ, GA and HEN for treating hepatic steatosis. Three compounds were identified as three factors, each having three dose levels (Table 3 and Table 4). IBM SPSS Statistics 27 was used to design the experiments and analyze the results.

### 4.10. Immunoblotting

Cells were lysed in 2× Laemmli buffer and boiled at 95 °C for 5 min. The homogenates were centrifuged at 12,000 rpm, and the supernatants were stored at −20 °C before use. Protein samples were separated by SDS-PAGE and blotted onto nitrocellulose membranes. After blocking in 5% non-fat milk in 1× TBS-T buffer for 2 h, the membranes were incubated with primary antibodies overnight at 4 °C [27]. The primary antibodies used were as follows: anti-AMPK, anti-p-AMPK (Thr172), anti-Akt, anti-p-Akt (Ser473), anti-p-ACC1 (Ser79), anti-CPT1α, anti-p-FoxO1, anti-p-IRS1 (Tyr895), anti-IRS1 (the above antibodies were used at 1:1000; Cell Signaling Technology, Danvers, MA, USA), anti-SREBP-1c, anti-FAS, anti-FoxO1, anti-PPARα, anti-ACOX1, anti-ACC1 (1:500; Santa Cruz Biotechnology, Dallas, TX, USA), anti-GLUT2, anti-HMGCS1 (1:1000, Proteintech, Wuhan, China) and anti-SREBP2 (1:1000, Thermo Fisher, Waltham, MA, USA). Subsequently, the membranes were incubated with the respective secondary antibodies (1:10,000, Thermo Fisher, Waltham, MA, USA) for 2 h at room temperature. The ChemiDoc Imaging System (Bio-Rad, Berkeley, CA, USA) was used for the visualization of immunoblots.

### 4.11. Statistical Analysis

All quantitative data are presented as the mean ± standard error of the mean (S.E.M.) from a minimum of three independent biological replicates (*n* = 3). Statistical analyses were performed using GraphPad Prism software (version 9.0). For comparisons among multiple groups, one-way analysis of variance (ANOVA) was employed, followed by Dunnett’s multiple comparisons test when comparing the treatment groups to a single control group. Differences were considered statistically significant at * *p* < 0.05, ** *p* < 0.01, and *** *p* < 0.001.

## 5. Conclusions

In conclusion, we provided new insights that ECD can ameliorate both NAFLD and IR in vitro, by activating the AMPK signaling pathway. We also identified the optimal combination of the main active compounds in ECD that can most effectively reduce lipid accumulation. However, the compound combination was not as good as the ECD formula, providing evidence for the superior synergistic effects of the herbs in ECD.

## Figures and Tables

**Table 1:** Orthogonal design analysis of the effects of compound combinations in reducing TG accumulation.

Group	A: LQ (μM)	B: GA (μM)	C: HEN (μM)	TG (Relative)
SM1	50	50	50	1.074
SM2	50	100	200	0.982
SM3	50	200	100	1.056
SM4	100	50	100	0.923
SM5	100	100	50	0.810
SM6	100	200	200	0.799
SM7	200	50	200	0.893
SM8	200	100	100	0.831
SM9	200	200	50	0.903
K1	3.112	2.890	2.714	
K2	2.531	2.695	2.882	
K3	2.627	2.685	2.684	
R	0.194	0.068	0.066	

The K-value represents the summation of TG (relative) values corresponding to a specific level of a factor in an orthogonal array. The R-value reflects the maximum variation in k-value (K/count of the level) across different levels of a factor. A higher R-value signifies its dominant influence in the system.

**Table 2:** The compounds and their quantities in ECD.

* Herbs	Place of Origin	Lot Number	Quantity
PT	Guizhou, China	20210801	15 g
CR	Guangdong, China	190601	15 g
PC	Hunan, China	201101	9 g
GU	Gansu, China	191005341	4.5 g
ZO	Guangdong, China	202108	7 pieces
PM	Sichuan, China	191001C085	1 piece

* *Pinellia ternata* (Thunb.) Breit. (PT), *Citrus reticulata* Blanco (CR), *Poria cocos* (Schwan.) Wolf. (PC), *Glycyrrhiza uralensis* Fisch. (GU), *Zingiber officinale* (Willd.) Rosc. (ZO) and *Prunus mume* (Sieh.) Sieb. et Zucc. (PM).

**Table 3:** The three-factor and three-level orthogonal experimental design.

	Factor	A: LQ	B: GA	C: HEN
Level				
1		50 μM	50 μM	50 μM
2		100 μM	100 μM	100 μM
3		200 μM	200 μM	200 μM

In the design of the orthogonal experiment, LQ, GA and HEN were considered as the three factors. Three concentrations of each compound were chosen as three levels.

**Table 4:** Orthogonal experimental design.

Group	A: LQ (μM)	B: GA (μM)	C: HEN (μM)
SM1	50	50	50
SM2	50	100	200
SM3	50	200	100
SM4	100	50	100
SM5	100	100	50
SM6	100	200	200
SM7	200	50	200
SM8	200	100	100
SM9	200	200	50

SM (substance mixture) 1–9 are the representative combinations of LQ, GA and HEN, based on L9(3)3 orthogonal experimental design by SPSS Statistics 27.

## Data Availability

Data is contained within the article and Supplementary Material.

## Supplementary Materials

The following supporting information can be downloaded at: https://www.mdpi.com/article/10.3390/ph18111707/s1, Figure S1: The determination of the content of hesperidin in ECD extract by HPLC; Figure S2: The effects of ECD on cell viability in HepG2 NAFLD model cells; Figure S3A. The herb-compound-target network of ECD; Figure S3B. Venn diagram of overlapped targets between ECD and diseases; Figure S3C. GO enrichment result of ECD acting on NAFLD and IR; Figure S4: The structural images of molecular docking; Figure S5: The effects of main compounds in ECD in reducing TG and TC contents; Table S1: Active compounds and potential targets of ECD collected in network pharmacology analysis; Table S2: Common targets between ECD-related targets and diseases-related targets; Table S3: The binding energy between hub targets and main compounds; Table S4: Orthogonal design variance analysis of the effects of compound combinations in reducing TG accumulation; Table S5: The calculation of the Combination Index (CI) of SM6.

## Abbreviations

The following abbreviations are used in this manuscript:

ACC1	Acetyl-CoA Carboxylase 1
ACOX-1	peroxisomal acyl-coenzyme A oxidase 1
AMPK	AMP-activated protein kinase
Akt	protein kinase B
CPT-1α	carnitine palmitoyltransferase 1α
ECD	*ErChen* decoction
FAS	fatty acid synthase
FoxO1	Forkhead box protein O1
GA	glycyrrhizic acid
GLUT2	glucose transporter type 2
HEN	hesperidin
HMGCS1	3-Hydroxy-3-Methylglutaryl-CoA Synthase 1
IR	insulin resistance
LQ	liquiritin
NAFLD	Non-alcoholic fatty liver disease
PPARα	peroxisome proliferator-activated receptor α
SREBP-1c	sterol regulatory element-binding protein 1c
SREBP-2	sterol regulatory element-binding protein 2
TCM	traditional Chinese medicine
T2DM	type II diabetes
