# Supplementary material for: The ErChen Decoction and Its Active Compounds Ameliorate Non-Alcoholic Fatty Liver Disease Through Activation of the AMPK Signaling Pathway
Source: Pharmaceuticals (Basel). 2025 Nov 11;18(11):1707. doi: 10.3390/ph18111707 (PMC12655137; doi:10.3390/ph18111707)
Supplement: Supplementary file 1 [file pharmaceuticals-18-01707-s001.zip › Supplementary Figure S1.pdf]

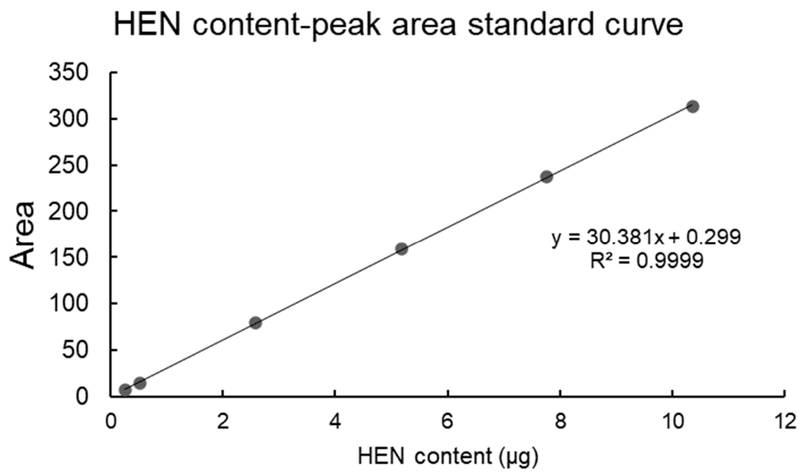

Chemical	Peak Area		
Hesperidin	114.2508	114.345	114.4188

Supplementary Figure S1. The determination of the content of hesperidin in ECD extract by HPLC

The X-axis is the contents of HEN (0.259 µg, 0.518 µg, 2.59 µg, 5.18 µg, 7.77 µg, 10.39 µg). The Y-axis is the average peak area. By evaluating the peak area of hesperidin (HEN) in the ECD sample, we estimated its content to be 5.63 mg (HEN) / g (ECD).
