# Supplementary material for: The ErChen Decoction and Its Active Compounds Ameliorate Non-Alcoholic Fatty Liver Disease Through Activation of the AMPK Signaling Pathway
Source: Pharmaceuticals (Basel). 2025 Nov 11;18(11):1707. doi: 10.3390/ph18111707 (PMC12655137; doi:10.3390/ph18111707)
Supplement: Supplementary file 1 [file pharmaceuticals-18-01707-s001.zip › Supplementary Figure S2.pdf]

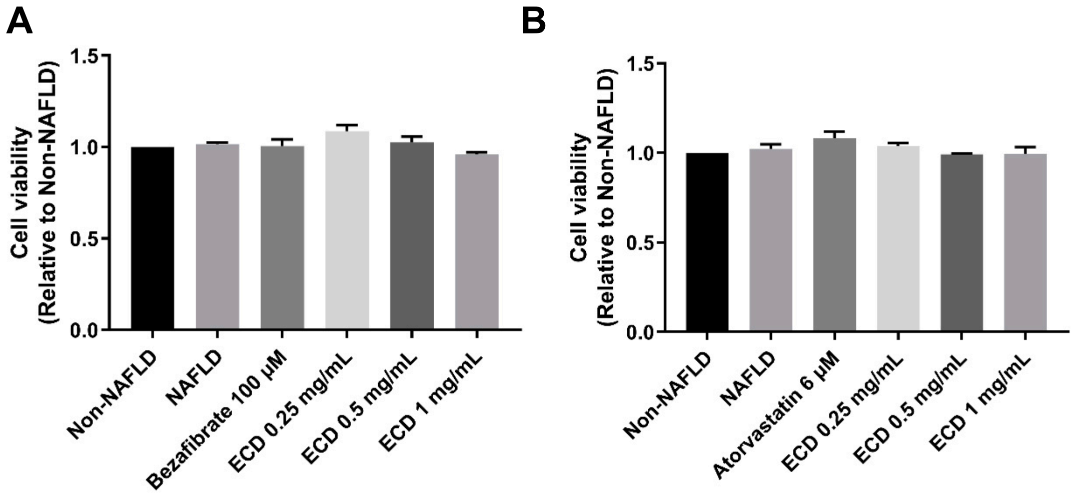

Supplementary Figure S2. The effects of ECD on cell viability in HepG2 NAFLD model cells

ECD treatment did not affect cell viability significantly in HepG2 NAFLD cells. (**A**) HepG2 cells were treated with 0.05 mM OAPA mix together with ECD at three concentrations of ECD for 24 hrs first. The cells were then incubated with ECD in the absence of the OAPA mix for another 24 hrs. (**B**) The cells were pre-treated with ECD for 24 hrs first. The cells were then incubated with 0.2 mM OAPA mix and ECD for another 24 hrs. The cell viability was determined using WST-1 assay.
