# Supplementary material for: The ErChen Decoction and Its Active Compounds Ameliorate Non-Alcoholic Fatty Liver Disease Through Activation of the AMPK Signaling Pathway
Source: Pharmaceuticals (Basel). 2025 Nov 11;18(11):1707. doi: 10.3390/ph18111707 (PMC12655137; doi:10.3390/ph18111707)
Supplement: Supplementary file 1 [file pharmaceuticals-18-01707-s001.zip › Supplementary Figure S3.pdf]

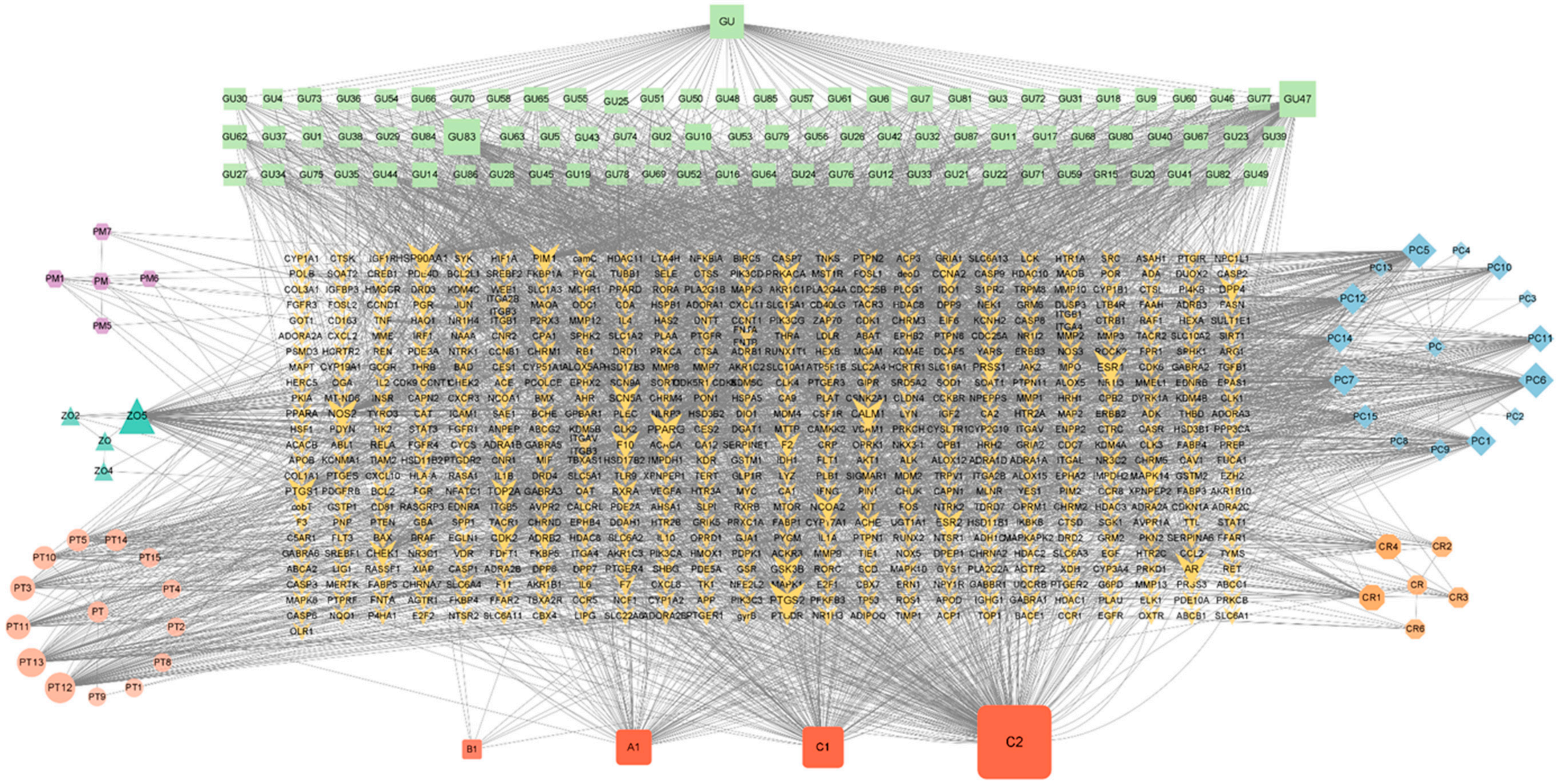

Supplementary Figure S3A. The herb-compound-target network of ECD.

The network comprises 71 nodes and 361 edges. The polygons at the periphery represent the single herbs and bioactive compounds in ECD. The yellow V-shape represent potential targets of ECD. A1 is Stigmasterol, the common ingredient between PT, ZO and PM. B1 is Sitosterol, the common ingredient between CR and GU. C1 is Kaempferol and C2 is Quercetin, the common ingredients between GU and PM.

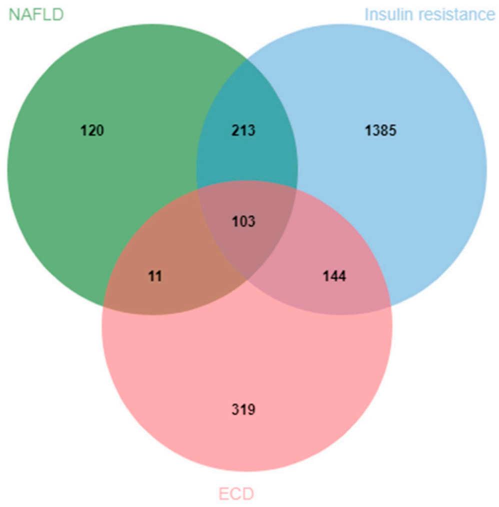

Supplementary Figure S3B. Venn diagram of overlapped targets between ECD and diseases

The green and blue circles indicate NAFLD and IR targets respectively. The pink circle indicates targets of the ECD compounds. The intersection targets are of a total of 258 (11+103+144) at the center.

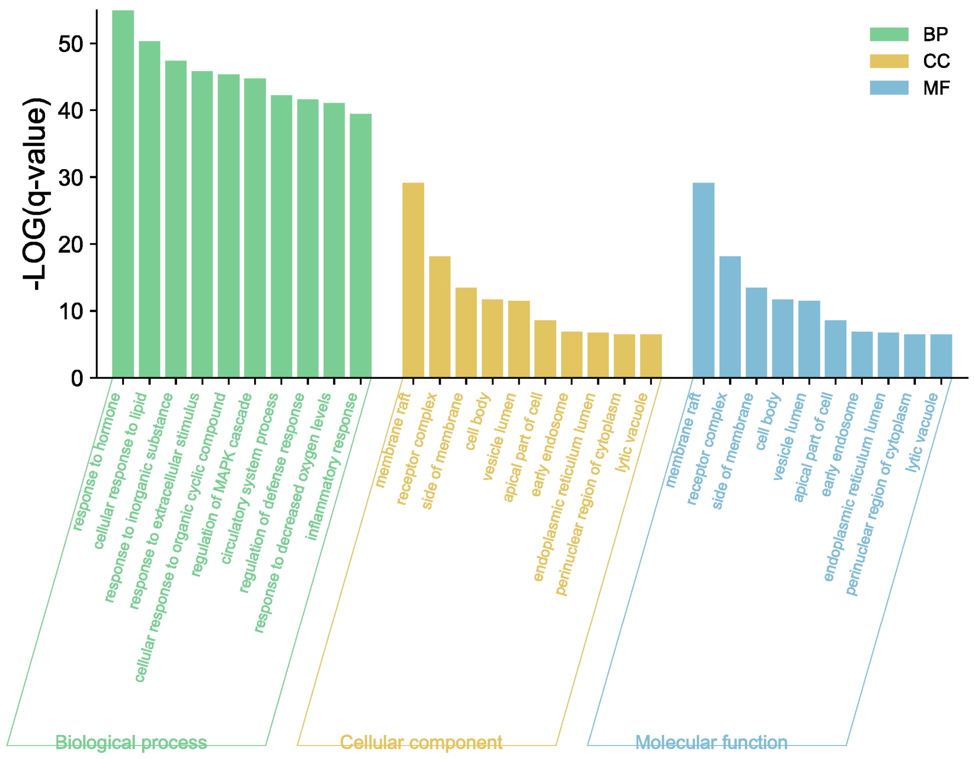

Supplementary Figure S3C. GO enrichment result of ECD acting on NAFLD and IR

Biological Process (BP), Cellular Component (CC), and Molecular Function (MF) are the items chosen to reflect the functions of potential targets of ECD acting on NAFLD and IR.
