# Supplementary material for: The ErChen Decoction and Its Active Compounds Ameliorate Non-Alcoholic Fatty Liver Disease Through Activation of the AMPK Signaling Pathway
Source: Pharmaceuticals (Basel). 2025 Nov 11;18(11):1707. doi: 10.3390/ph18111707 (PMC12655137; doi:10.3390/ph18111707)
Supplement: Supplementary file 1 [file pharmaceuticals-18-01707-s001.zip › Supplementary Figure S4.pdf]

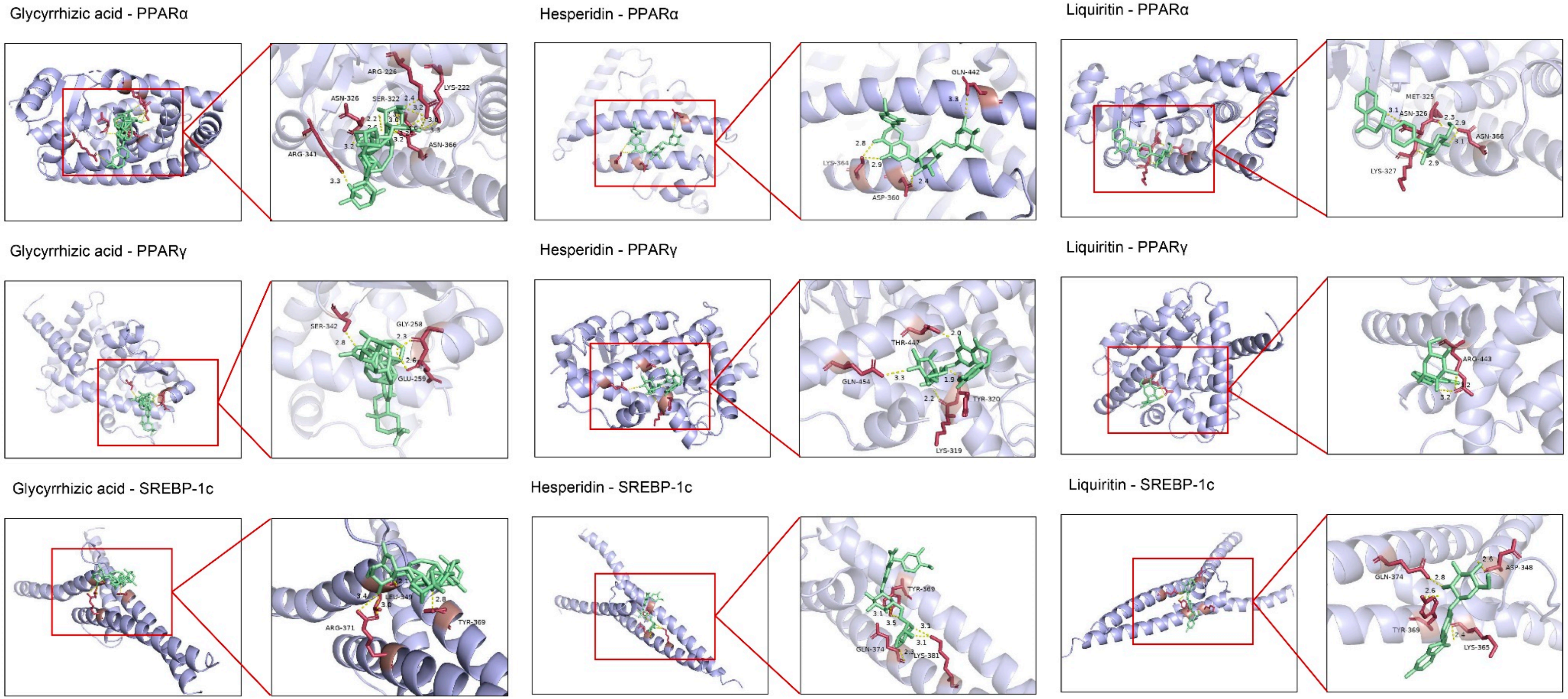

Supplementary Figure S4. The structural images of molecular docking

The images indicate the predicted situations that HEN, GA and LQ interact with PPARα, PPARγ and SREBP-1c, respectively. The yellow dotted lines indicate the hydrogen bonds. The green rodlike molecules represent compounds. The purple screwed molecules represent proteins. The red parts represent the amino acids that form hydrogen bonds with compounds.
