# Supplementary material for: The ErChen Decoction and Its Active Compounds Ameliorate Non-Alcoholic Fatty Liver Disease Through Activation of the AMPK Signaling Pathway
Source: Pharmaceuticals (Basel). 2025 Nov 11;18(11):1707. doi: 10.3390/ph18111707 (PMC12655137; doi:10.3390/ph18111707)
Supplement: Supplementary file 1 [file pharmaceuticals-18-01707-s001.zip › Supplementary Figure S5.pdf]

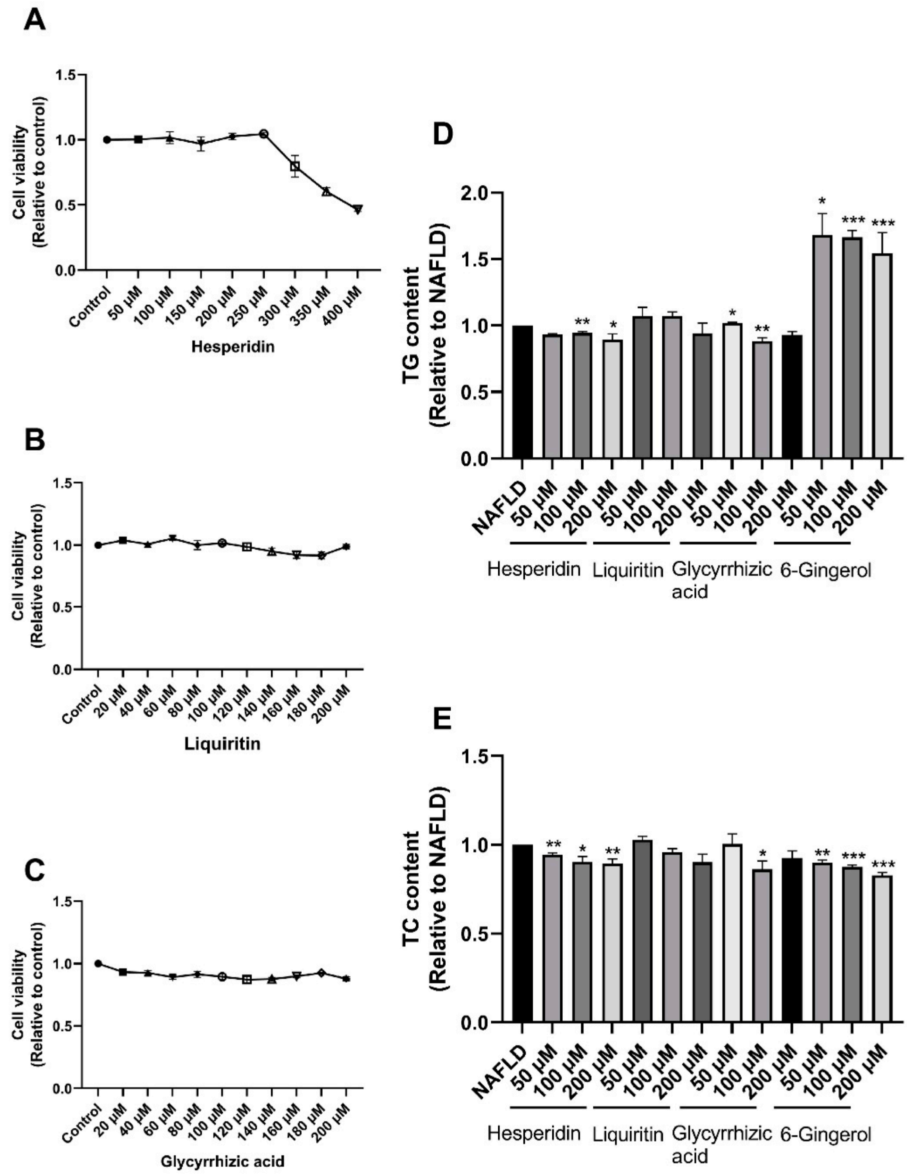

Supplementary Figure S5. The effects of main compounds in ECD in reducing TG and TC contents

(**A-C**) The HepG2 cells were treated with the candidate drugs for 48 hrs and the cell viability was measured by the CCK-8 assay. (**D**) For TG measurement, HepG2 cells were treated with 0.05 mM OAPA mix and the candidate drugs for 24 hrs. The cells were then incubated with ECD in the absence of the OAPA mix for another 24 hrs. (**E**) For TC measurement, the cells were pretreated with the drugs for 24 hrs. The cells were then treated with 0.2 mM OAPA and the candidate drugs for another 24 hrs. The levels of TG and TC were determined following the instructions of the corresponding assay kits. The results are expressed as the mean ± S.E.M. ($n \geq 3$). *, $p < 0.05$; **, $p < 0.01$ and ***, $p < 0.001$ vs. NAFLD model.
