# Supplementary material for: The ErChen Decoction and Its Active Compounds Ameliorate Non-Alcoholic Fatty Liver Disease Through Activation of the AMPK Signaling Pathway
Source: Pharmaceuticals (Basel). 2025 Nov 11;18(11):1707. doi: 10.3390/ph18111707 (PMC12655137; doi:10.3390/ph18111707)
Supplement: Supplementary file 1 [file pharmaceuticals-18-01707-s001.zip › Supplementary Table S1.pdf]

**Supplementary Table S1**

| 577 targets of ECD | 130 active compounds of ECD |
|---|---|
| PTGS1 | gondoic acid |
| NCOA2 | 10,13-eicosadienoic |
| AR | baicalein |
| PTGS2 | (3S,6S)-3-(benzyl)-6-(4-hydroxybenzyl)piperazine-2,5-quinone |
| HSP90AA1 | coniferin |
| PRKACA | beta-sitosterol |
| DPP4 | Stigmasterol |
| PIK3CG | 24-Ethylcholest-4-en-3-one |
| PDE3A | Cycloartenol |
| PRSS1 | Cavidine |
| NCOA1 | Pedatisectine F |
| CALM1 | L-Tyrosyl-L-alanine |
| RELA | Pinellic acid |
| AKT1 | HMF |
| BCL2 | NCA |
| FOS | naringenin |
| BAX | 5,7-dihydroxy-2-(3-hydroxy-4-methoxyphenyl)chroman-4-one |
| MMP9 | Citromitin |
| CASP3 | nobiletin |
| TP53 | sitosterol |
| HIF1A | hesperidin |
| FOSL1 | dehydroeburicoic acid |
| FOSL2 | ergosta-7,22E-dien-3beta-ol |
| CDK1 | Ergosterol peroxide |
| CCNB1 | trametenolic acid |
| MPO | 3beta-Hydroxy-24-methylene-8-lanostene-21-oic acid (Eburicoic Acid) |
| AHR | (2R)-2-[(5R,10S,13R,14R,16R,17R)-16-hydroxy-3-keto-4,4,10,13,14-pentamethyl-1,2,5,6,12,15,16,17-octahydrocyclopenta[a]phenanthren-17-yl]-5-isopropyl-hex-5-enoic acid |
| IGF2 | poricoic acid C |
| CYCS | Cerevisterol |
| ALOX12 | hederagenin |
| NFATC1 | 7,9(11)-dehydropachymic acid |
| TDRD7 | pachymic acid |
| EGLN1 | (2R)-2-[(3S,5R,10S,13R,14R,16R,17R)-3,16-dihydroxy-4,4,10,13,14-pentamethyl-2,3,5,6,12,15,16,17-octahydro-1H-cyclopenta[a]phenanthren-17-yl]-5-isopropyl-hex-5-enoic acid |

| | |
|---|---|
| NOX5 | (2R)-2-[(3S,5R,10S,13R,14R,16R,17R)-3,16-dihydroxy-4,4,10,13,14-pentamethyl-2,3,5,6,12,15,16,17-octahydro-1H-cyclopenta[a]phenanthren-17-yl]-6-methylhept-5-enoic acid |
| FABP5 | Poricoic acid A |
| APOD | Poricoic acid B |
| deoD | Inermine |
| CHRM3 | DFV |
| CHRM1 | Mairin |
| ESR1 | Glycyrol |
| SCN5A | Jaranol |
| CA2 | Medicarpin |
| ADRA1B | isorhamnetin |
| ADRA1D | Lupiwighteone |
| TOP2A | 7-Methoxy-2-methyl isoflavone |
| OPRM1 | formononetin |
| CDK2 | Calycosin |
| DPEP1 | kaempferol |
| CHRNA7 | (2S)-2-[4-hydroxy-3-(3-methylbut-2-enyl)phenyl]-8,8-dimethyl-2,3-dihydropyrano[2,3-f]chromen-4-one |
| IGHG1 | euchrenone |
| PIM1 | glyasperin B |
| CCNA2 | glyasperin F |
| PGR | Glyasperin C |
| KCNH2 | Isotrifoliol |
| DRD1 | (E)-1-(2,4-dihydroxyphenyl)-3-(2,2-dimethylchromen-6-yl)prop-2-en-1-one |
| GABRA2 | kanzonols W |
| CHRM4 | (2S)-6-(2,4-dihydroxyphenyl)-2-(2-hydroxypropan-2-yl)-4-methoxy-2,3-dihydrofuro[3,2-g]chromen-7-one |
| HTR2A | Semilicoisoflavone B |
| GABRA5 | Glepidotin A |
| ADRA1A | Glepidotin B |
| GABRA3 | Phaseolinisoflavan |
| CHRM2 | Glypallichalcone |
| ADRB2 | 8-(6-hydroxy-2-benzofuranyl)-2,2-dimethyl-5-chromenol |
| CHRNA2 | Licochalcone B |
| SLC6A4 | licochalcone G |
| GABRA1 | 3-(2,4-dihydroxyphenyl)-8-(1,1-dimethylprop-2-enyl)-7-hydroxy-5-methoxy-coumarin |
| camC | Licoricone |
| CASP9 | Gancaonin A |
| JUN | Gancaonin B |
| CASP8 | 3-(3,4-dihydroxyphenyl)-5,7-dihydroxy-8-(3-methylbut-2-enyl)chromone |
| PRKCA | 5,7-dihydroxy-3-(4-methoxyphenyl)-8-(3-methylbut-2-enyl)chromone |

| | |
|---|---|
| TGFB1 | 2-(3,4-dihydroxyphenyl)-5,7-dihydroxy-6-(3-methylbut-2-enyl)chromone |
| PON1 | Glycyrin |
| MAP2 | Licocoumarone |
| NR3C2 | Licoisoflavone |
| RXRA | Licoisoflavone B |
| ADRA2A | licoisoflavanone |
| SLC6A2 | shinpterocarpin |
| SLC6A3 | (E)-3-[3,4-dihydroxy-5-(3-methylbut-2-enyl)phenyl]-1-(2,4-dihydroxyphenyl)prop-2-en-1-one |
| AKR1B1 | licopyranocoumarin |
| PLAU | 3,22-Dihydroxy-11-oxo-delta(12)-oleanene-27-alpha-methoxycarbonyl-29-oic acid |
| LTA4H | Glyzaglabrin |
| MAOB | Glabridin |
| MAOA | Glabranin |
| CTRB1 | Glabrene |
| ADRB1 | Glabrone |
| F10 | 1,3-dihydroxy-9-methoxy-6-benzofurano[3,2-c]chromenone |
| CHRM5 | 1,3-dihydroxy-8,9-dimethoxy-6-benzofurano[3,2-c]chromenone |
| HTR3A | Eurycarpin A |
| ADRA2C | Sigmoidin-B |
| OPRD1 | (2R)-7-hydroxy-2-(4-hydroxyphenyl)chroman-4-one |
| HTR2C | (2S)-7-hydroxy-2-(4-hydroxyphenyl)-8-(3-methylbut-2-enyl)chroman-4-one |
| F7 | Isoglycyrol |
| PDE10A | Isolicoflavonol |
| ADA | HMO |
| CDA | 1-Methoxyphaseollidin |
| HEXA | Quercetin der. |
| HEXB | 3'-Hydroxy-4'-O-Methylglabridin |
| OGA | licochalcone a |
| CA9 | 3'-Methoxyglabridin |
| ADK | 2-[(3R)-8,8-dimethyl-3,4-dihydro-2H-pyrano[6,5-f]chromen-3-yl]-5-methoxyphenol |
| CA12 | Inflacoumarin A |
| FUCA1 | icos-5-enoic acid |
| CA1 | Kanzonol F |
| PNP | 6-prenylated eriodictyol |
| PYGM | 7,2',4'-trihydroxy − 5-methoxy-3 − arylcoumarin |
| TYMS | 7-Acetoxy-2-methylisoflavone |
| TK1 | 8-prenylated eriodictyol |
| CASP6 | gadelaidic acid |
| CASP7 | Vestitol |

| | |
|---|---|
| CASP2 | Gancaonin G |
| ADORA1 | Gancaonin H |
| ADORA2A | Licoagrocarpin |
| KDM4E | Glyasperins M |
| CDK9 CCNT1 | Glycyrrhiza flavonol A |
| CSNK2A1 | Licoagroisoflavone |
| ADORA3 | 18α-hydroxyglycyrrhetic acid |
| F2 | Odoratin |
| PTGES | Phaseol |
| PIN1 | Xambioona |
| GBA | dehydroglyasperins C |
| PTPN1 | quercetin |
| LIG1 | 6-methylgingediacetate2 |
| MAPK1 | poriferast-5-en-3beta-ol |
| P4HA1 | Dihydrocapsaicin |
| PTPN2 | (2R)-5,7-dihydroxy-2-(4-hydroxyphenyl)chroman-4-one |
| SLC15A1 | campest-5-en-3beta-ol |
| MME | Methyl arachidonate |
| YARS | CLR |
| ACE | |
| CCKBR | |
| PDYN | |
| OPRK1 | |
| HLA-A | |
| CAPN1 | |
| TACR1 | |
| NTSR1 | |
| XIAP | |
| CBX7 | |
| NTSR2 | |
| GRIA1 | |
| HMGCR | |
| TACR3 | |
| NAAA | |
| NOS3 | |
| EPHX2 | |
| MMP1 | |
| STAT3 | |
| CPB1 | |
| IL1B | |
| MLNR | |
| CHRND | |
| MAPKAPK2 | |

| | |
|---|---|
| CASP1 | |
| ERN1 | |
| CBX4 | |
| LCK | |
| KDM4C | |
| DPP9 | |
| CPA1 | |
| FPR1 | |
| SORT1 | |
| CPB2 | |
| ANPEP | |
| GRIA2 | |
| SLC5A1 | |
| PIM2 | |
| DDAH1 | |
| GRM2 | |
| DPP8 | |
| GABBR1 | |
| SLC22A6 | |
| SRC | |
| SLC1A3 | |
| GRM6 | |
| SLC1A2 | |
| ARG1 | |
| KDM5C | |
| KDM4B | |
| KDM5B | |
| KDM4A | |
| XPNPEP1 | |
| XPNPEP2 | |
| GRIK5 | |
| PTGER4 | |
| PTGER2 | |
| PTGER1 | |
| PTGIR | |
| PPARD | |
| LTB4R | |
| PTGFR | |
| CYP19A1 | |
| PTGER3 | |
| PPARA | |
| PPARG | |
| FABP4 | |

| | |
|---|---|
| TERT | |
| FABP3 | |
| FABP1 | |
| SERPINA6 | |
| SHBG | |
| G6PD | |
| PTGDR | |
| NPC1L1 | |
| RORA | |
| FAAH | |
| FNTA FNTB | |
| GCGR | |
| GIPR | |
| NOS2 | |
| CES2 | |
| PREP | |
| GLP1R | |
| AKR1B10 | |
| CYP17A1 | |
| FFAR1 | |
| PDE5A | |
| MMP13 | |
| NR1H3 | |
| POLB | |
| PLA2G1B | |
| ADRB3 | |
| THRA | |
| THRB | |
| SRD5A2 | |
| TYRO3 | |
| DGAT1 | |
| LYZ | |
| cobT | |
| ACHE | |
| GABRA6 | |
| PRSS3 | |
| MAPK3 | |
| FASN | |
| LDLR | |
| BAD | |
| SOD1 | |
| CAT | |
| MTTP | |

| | |
|---|---|
| APOB | |
| PLB1 | |
| GSTP1 | |
| UGT1A1 | |
| SREBF1 | |
| GSR | |
| ABCC1 | |
| ADIPOQ | |
| SOAT2 | |
| AKR1C1 | |
| GOT1 | |
| ABAT | |
| CES1 | |
| SOAT1 | |
| CAMKK2 | |
| KCNMA1 | |
| ESR2 | |
| CHEK1 | |
| GSK3B | |
| MAPK8 | |
| TIMP1 | |
| CREB1 | |
| PLA2G4A | |
| CD163 | |
| EPHB2 | |
| HSD11B1 | |
| NR3C1 | |
| RORC | |
| PTPRF | |
| ACP1 | |
| PDE4D | |
| CDC25B | |
| TNF | |
| PTPN6 | |
| CYP51A1 | |
| PTPN11 | |
| SAE1 | |
| PRKCH | |
| HSD11B2 | |
| SIGMAR1 | |
| CD81 | |
| IKBKB | |
| VDR | |

| | |
|---|---|
| SCD | |
| FNTA | |
| TOP1 | |
| BCL2L1 | |
| PLCG1 | |
| HAO1 | |
| CDC25A | |
| ALOX5AP | |
| ALOX5 | |
| BCHE | |
| CYSLTR1 | |
| PTGDR2 | |
| MDM2 | |
| RASGRP3 | |
| BACE1 | |
| SREBF2 | |
| IDO1 | |
| NR1H4 | |
| NR1I2 | |
| HSD17B3 | |
| NR1I3 | |
| GPBAR1 | |
| CYP2C19 | |
| CNR1 | |
| TRPV1 | |
| EDNRA | |
| CTSD | |
| FDFT1 | |
| AGTR1 | |
| CCR1 | |
| FKBP1A | |
| S1PR2 | |
| ITGB1 | |
| MMP2 | |
| MMP8 | |
| MMEL1 | |
| PLEC | |
| ITGAV | |
| MIF | |
| ITGA2B | |
| ITGB5 | |
| SGK1 | |
| FKBP5 | |

| | |
|---|---|
| MAPK10 | |
| FKBP4 | |
| EGFR | |
| PYGL | |
| TRPM8 | |
| P2RX3 | |
| MMP3 | |
| MMP7 | |
| EDNRB | |
| ENPP2 | |
| TBXAS1 | |
| ALOX15 | |
| AVPR2 | |
| AVPR1A | |
| ITGA4 | |
| GYS1 | |
| FFAR2 | |
| OXTR | |
| TACR2 | |
| AKR1C2 | |
| EPAS1 | |
| NLRP3 | |
| CTRC | |
| HSD17B2 | |
| EPHA2 | |
| CALCRL | |
| AKR1C3 | |
| PLA2G2A | |
| TBXA2R | |
| ABCB1 | |
| MMP10 | |
| MMP12 | |
| SLC10A2 | |
| SLC10A1 | |
| CASR | |
| IMPDH1 | |
| IMPDH2 | |
| PDE2A | |
| TTL | |
| PIK3CA | |
| AGTR2 | |
| CCR5 | |
| SLC6A1 | |

| | |
|---|---|
| TUBB1 | |
| PKIA | |
| KDR | |
| MAPK14 | |
| XDH | |
| NCF1 | |
| OLR1 | |
| IL4 | |
| SIRT1 | |
| ATP5F1B | |
| MT-ND6 | |
| HSD3B2 | |
| HSD3B1 | |
| AHSA1 | |
| STAT1 | |
| HMOX1 | |
| CYP3A4 | |
| CYP1A2 | |
| CYP1A1 | |
| ICAM1 | |
| SELE | |
| VCAM1 | |
| CYP1B1 | |
| HAS2 | |
| PSMD3 | |
| SLC2A4 | |
| INSR | |
| DIO1 | |
| PPP3CA | |
| GSTM1 | |
| GSTM2 | |
| SLPI | |
| RXRB | |
| APP | |
| CTSK | |
| CTSS | |
| CTSL | |
| DNTT | |
| HCRTR2 | |
| HCRTR1 | |
| CAPN2 | |
| MDM4 | |
| ACACB | |

| | |
|---|---|
| IDH1 | |
| EZH2 | |
| MTOR | |
| C5AR1 | |
| PDPK1 | |
| PRKD1 | |
| PIK3C3 | |
| IGF1R | |
| PIK3CD | |
| PI4KB | |
| CCNT1 | |
| REN | |
| SLC16A1 | |
| CXCR3 | |
| CSF1R | |
| ABL1 | |
| FLT1 | |
| PDGFRB | |
| KIT | |
| FLT3 | |
| ACKR3 | |
| RET | |
| PFKFB3 | |
| SYK | |
| MST1R | |
| FGFR3 | |
| ZAP70 | |
| NTRK1 | |
| JAK2 | |
| BMX | |
| LYN | |
| FGFR4 | |
| ALK | |
| FGR | |
| NTRK2 | |
| BRAF | |
| TIE1 | |
| MERTK | |
| ROS1 | |
| CCND1 | |
| EIF6 | |
| RB1 | |
| CDK6 | |

| | |
|---|---|
| ITGAV ITGB3 | |
| ITGAL | |
| OAT | |
| HDAC3 | |
| HDAC6 | |
| HDAC2 | |
| F11 | |
| HDAC1 | |
| HDAC11 | |
| HDAC10 | |
| ITGA2B ITGB3 | |
| ITGB1 ITGA4 | |
| CTSA | |
| VEGFA | |
| CDKN1A | |
| IL10 | |
| EGF | |
| IL6 | |
| ELK1 | |
| NFKBIA | |
| POR | |
| ODC1 | |
| RAF1 | |
| RUNX1T1 | |
| HERC5 | |
| HSPA5 | |
| ERBB2 | |
| ACACA | |
| CAV1 | |
| MYC | |
| F3 | |
| GJA1 | |
| CCL2 | |
| CXCL8 | |
| PRKCB | |
| BIRC5 | |
| DUOX2 | |
| HSPB1 | |
| SULT1E1 | |
| MGAM | |
| IL2 | |
| PLAT | |
| THBD | |

| | |
|---|---|
| SERPINE1 | |
| COL1A1 | |
| IFNG | |
| PTEN | |
| IL1A | |
| ABCA2 | |
| NFE2L2 | |
| NQO1 | |
| TNKS | |
| COL3A1 | |
| CXCL11 | |
| CXCL2 | |
| DCAF5 | |
| CHEK2 | |
| CLDN4 | |
| HSF1 | |
| CRP | |
| CXCL10 | |
| CHUK | |
| SPP1 | |
| RUNX2 | |
| RASSF1 | |
| E2F1 | |
| E2F2 | |
| ACP3 | |
| IGFBP3 | |
| CD40LG | |
| IRF1 | |
| ERBB3 | |
| PCOLCE | |
| NPEPPS | |
| HK2 | |
| NKX3-1 | |
| RASA1 | |
| ADH1C | |
| SLC6A11 | |
| SLC6A13 | |
| DRD3 | |
| HDAC8 | |
| CNR2 | |
| ROCK2 | |
| SCN9A | |
| HRH1 | |

| | |
|---|---|
| FGFR1 | |
| HRH2 | |
| DRD4 | |
| YES1 | |
| EPHB4 | |
| DRD2 | |
| ADRA2B | |
| NPY1R | |
| LIPG | |
| HTR1A | |
| WEE1 | |
| NEK1 | |
| SPHK2 | |
| SPHK1 | |
| CDK5R1 CDK5 | |
| DYRK1A | |
| ABCG2 | |
| CDC7 | |
| TLR9 | |
| CCR8 | |
| HTR2B | |
| PKN2 | |
| DPP7 | |
| UQCRB | |
| ADORA2B | |
| ASAH1 | |
| MCHR1 | |
| DUSP3 | |
| PLAA | |
| MAPT | |
| CLK4 | |
| CLK1 | |
| CLK2 | |
| CLK3 | |
| PRXC1A | |
| gyrB | |
| TIAM2 | |
