# Supplementary material for: The ErChen Decoction and Its Active Compounds Ameliorate Non-Alcoholic Fatty Liver Disease Through Activation of the AMPK Signaling Pathway
Source: Pharmaceuticals (Basel). 2025 Nov 11;18(11):1707. doi: 10.3390/ph18111707 (PMC12655137; doi:10.3390/ph18111707)
Supplement: Supplementary file 1 [file pharmaceuticals-18-01707-s001.zip › Supplementary Table S2.pdf]

Supplementary Table S2

PTGS2
ESR1
AR
PPARG
CALM1
HSP90AA1
NOS2
PIM1
NCOA2
PTGS1
ESR2
PRSS1
GSK3B
CDK2
SCN5A
CCNA2
CHEK1
MAPK14
DPP4
TOP2A
F2
PRKACA
ADRB2
RXRA
PGR
ADRA1B
ACHE
CHRM1
PIK3CG
KCNH2
F7
NCOA1
PDE3A
NOS3
NR3C2
PTPN1
CHRM3
KDR
GABRA1
SLC6A3
SLC6A4
HMGCR

IGHG1
MAOB
CASP3
CHRM2
PPARA
PPARD
TNF
ALOX5AP
