# Supplementary material for: The ErChen Decoction and Its Active Compounds Ameliorate Non-Alcoholic Fatty Liver Disease Through Activation of the AMPK Signaling Pathway
Source: Pharmaceuticals (Basel). 2025 Nov 11;18(11):1707. doi: 10.3390/ph18111707 (PMC12655137; doi:10.3390/ph18111707)
Supplement: Supplementary file 1 [file pharmaceuticals-18-01707-s001.zip › Supplementary Table S3.pdf]

Supplementary Table S3. The binding energy between hub targets and main compounds

Targets	Compounds	Binding Energy (kcal/mol)	Targets	Compounds	Binding Energy (kcal/mol)	Targets	Compounds	Binding Energy (kcal/mol)
PPARα	Glycyrrhizic acid	-9.5	PPARγ	Glycyrrhizic acid	-10.5	SREBP-1	Glycyrrhizic acid	-9.3
	beta-Sitosterol	-8.3		Stigmasterol	-7.6		beta-Sitosterol	-7.7
	Stigmasterol	-8.3		Hesperidin	-7.5		Hesperidin	-7.3
	Quercetin	-7.7		Naringenin	-6.7		Stigmasterol	-6.9
	Hesperidin	-7.7		Liquiritin	-6.7		Kaempferol	-6.5
	Naringenin	-7.4		beta-Sitosterol	-6.6		Naringenin	-6.5
	Kaempferol	-7.2		Quercetin	-6.6		Liquiritin	-6.5
	Liquiritin	-7.1		Kaempferol	-5.9		Quercetin	-6.4
