# Supplementary material for: The ErChen Decoction and Its Active Compounds Ameliorate Non-Alcoholic Fatty Liver Disease Through Activation of the AMPK Signaling Pathway
Source: Pharmaceuticals (Basel). 2025 Nov 11;18(11):1707. doi: 10.3390/ph18111707 (PMC12655137; doi:10.3390/ph18111707)
Supplement: Supplementary file 1 [file pharmaceuticals-18-01707-s001.zip › Supplementary Table S4.pdf]

Supplementary Table S4. Orthogonal design variance analysis of the effects of compound combinations in reducing TG accumulation

	Sum of squares	df	Mean squares	F	Significance
A: LQ	0.065	2	0.032	241.276	0.004
B: GA	0.009	2	0.004	33.176	0.029
C: HEN	0.008	2	0.004	30.35	0.032
Error	0.000	9	0.000		
Sum	7.681	8			
