# Supplementary material for: The ErChen Decoction and Its Active Compounds Ameliorate Non-Alcoholic Fatty Liver Disease Through Activation of the AMPK Signaling Pathway
Source: Pharmaceuticals (Basel). 2025 Nov 11;18(11):1707. doi: 10.3390/ph18111707 (PMC12655137; doi:10.3390/ph18111707)
Supplement: Supplementary file 1 [file pharmaceuticals-18-01707-s001.zip › Supplementary Table S5.pdf]

Supplementary Table S5. The calculation of the Combination Index (CI) of SM6

	TG AVE (Relative to model)	TG reduction AVE
**LQ 100 μM**	1.07	-0.07
**GA 200 μM**	0.906	0.094
**HEN 200 μM**	0.898	0.102
**LQ 100+GA 200+HEN 200**	0.799	0.201
		CI=0.627

The calculation formula is CI = $\frac{E(A)}{E(AB)} + \frac{E(B)}{E(AB)}$, CI>1: antagonistic action, CI=1: additive effect, CI<1: synergistic effect.
